# A population genetics perspective on the evolutionary histories of three clonal, endemic, and dominant grass species of the Qinghai–Tibet Plateau: *Orinus* (Poaceae)

**DOI:** 10.1002/ece3.5186

**Published:** 2019-04-26

**Authors:** Yuping Liu, AJ Harris, Qingbo Gao, Xu Su, Zhumei Ren

**Affiliations:** ^1^ Key Laboratory of Medicinal Plant and Animal Resources of the Qinghai‐Tibet Plateau in Qinghai Province, School of Life Science Qinghai Normal University Xining China; ^2^ Key Laboratory of Physical Geography and Environmental Process in Qinghai Province, School of Life Science Qinghai Normal University Xining China; ^3^ Key Laboratory of Education Ministry of Environments and Resources in the Qinghai‐Tibet Plateau, School of Life Science Qinghai Normal University Xining China; ^4^ Department of Biology Oberlin College and Conservatory Oberlin Ohio; ^5^ Qinghai Provincial Key Laboratory of Crop Molecular Breeding, Northwest Institute of Plateau Biology Chinese Academy of Sciences Xining China; ^6^ School of Life Science Shanxi University Taiyuan China

**Keywords:** alpine grassland, amplified fragment length polymorphism, genetic variation, hybridization, population biology

## Abstract

We performed analyses of amplified fragment length polymorphism (AFLP) in order to characterize the evolutionary history of *Orinus* according to its population genetic structure, as well as to investigate putative hybrid origins of *O. intermedius* and to provide additional insights into relationships among species. The genus *Orinus* comprises three clonal grasses that are dominant species within xeric alpine grasslands of the Qinghai–Tibet Plateau (QTP). Here, we used eight selectively obtained primer pairs of *EcoR*I/*Mse*I to perform amplifications in 231 individuals of *Orinus* representing 48 populations and all three species. We compared our resulting data to genetic models of hybridization using a Bayesian algorithm within NewHybrids software. We determined that genetic variation in *Orinus* was 56.65% within populations while the among‐species component was 30.04% using standard population genetics statistics. Nevertheless, we detected that species of *Orinus* were clustered into three highly distinct genetic groups corresponding to classic species identities. Our results suggest that there is some introgression among species. Thus, we tested explicit models of hybridization using a Bayesian approach within NewHybrids software. However, *O. intermedius* likely derives from a common ancestor with *O. kokonoricus* and is probably not the result of hybrid speciation between *O. kokonoricus* and *O. thoroldii*. We suspect that recent isolation of species of *Orinus* in allopatry via vicariance may explain the patterns in diversity that we observed, and this is corroborated by a Mantel test that showed significant positive correlation between geographic and genetic distance (*r* = 0.05, *p* < 0.05). Recent isolation may explain why *Orinus* differs from many other clonal species by exhibiting the highest diversity within populations rather than among them.

## 1 INTRODUCTION

Genetic diversity is a particularly significant factor in the long‐term stability of plant populations (Hedrick, 2001; Jump, Marchant, & Peñuelas, 2009; Rahimmalek, Tabatabaei, Arzani, & Etemadi, 2009; Wang et al., 2007). For example, low genetic diversity of a population may both represent critical local adaptation and, simultaneously, limit overall evolutionary potential in the face of environmental disturbances (Cortés et al., 2014; Jump et al., 2009; Sedlacek et al., 2016, 2015). Therefore, knowledge of population genetic diversity is extremely important for recognizing conservation needs and developing sustainable strategies (Gordon, Sloop, Davis, & Cushman, 2012; Kaljund & Jaaska, 2010). Conservation of species is an urgent global issue, especially within biodiversity hotspots, such as the Qinghai–Tibet Plateau (QTP) and surrounding mountainous areas, which represent some of the highest priorities within temperate zones for conservation research and implementations (Beger et al., 2015; Maréchaux, Rodrigues, & Charpentier, 2016; Myers, Mittermeier, Mittermeier, Da Fonseca, & Kent, 2000).

The biodiversity of the QTP appears to be correlated with its complex, recent history of environmental change and its present‐day heterogeneous landscape. Environmental change and landscape heterogeneity are well‐known drivers of biodiversity according to classic ecological theory (Risser, 1987). In the present, the QTP exhibits substantial landscape heterogeneity; for example, its elevation range is from 3,000 to 5,000 m and represents a steep ecological gradient comprising diverse niches for a rich composition of species (Feng et al., 2017; Feng et al., 2017; Liu, Luo, Li, & Gao, 2017). With respect to environmental change, the QTP has undergone extreme ecological disturbances on an evolutionary timescale, especially rapid uplifts since the Miocene–Pliocene or Miocene–Quaternary epochs and subsequent climatic oscillations in the Quaternary (Liu, 2004; Liu, Gao, Chen, & Lu, 2002; Liu, Wang, Geng, et al., 2006; Liu, Wang, Wang, Hideaki, & Abbott, 2006; Liu et al., 2018, 2015; Shi, 2002; Shi, Li, & Li, 1998; Wen, Zhang, Nie, Zhong, & Sun, 2014; Zheng & Nat, 1998). The biodiversity within the QTP is reflected within its flora, which harbors ca. 9,000 vascular plant species of which more than 18% are endemic (Wu, 2008), including at least 20 endemic genera (Wu, Yang, & Fei, 1995).

Many recent studies have sought to address evolutionary diversification of plant species within the QTP and have especially used population genetics methods to elucidate patterns of diversity and distributions and better understand the underlying mechanisms (Liu, Wang, Geng, et al., 2006; Ren, Conti, & Salamin, 2015; Wen et al., 2014). Recently, Wen et al. (2014) reviewed current evidence of mechanisms of speciation on the QTP using exemplar species within diverse vascular plant families, especially of Asteraceae, Crassulaceae, Ericaceae, Orobanchaceae, and Papaveraceae. However, the mechanisms of speciation within alpine areas of the QTP (and beyond) remain poorly understood. These mechanisms likely include allopatric processes and, possibly, rapid genetic isolation due to increased mutation rates under high levels of ultraviolet light exposure (Davies, Savolainen, Chase, Moat, & Barraclough, 2004; Madriñán, Cortés, & Richardson, 2013; Willis, Bennett, & Birks, 2009). Within the QTP, studies of many plant species are needed to serve as models for diversification and speciation patterns and processes, especially to represent the numerous habits, life histories, environmental preferences, and other features of the rich botanical diversity of the region. Such studies are particularly urgent for regions, such as the alpine grasslands (Bowman, 2000; Li et al., 2014; Yi et al., 2011), that have become imperiled during the Anthropocene (Crutzen & Stoermer, 2000) especially due to climate change and pressures from intensive grazing by livestock (Han, Brierley, Cullum, & Li, 2016; Wilcox, Sorice, & Young, 2011).

**Figure 1:**
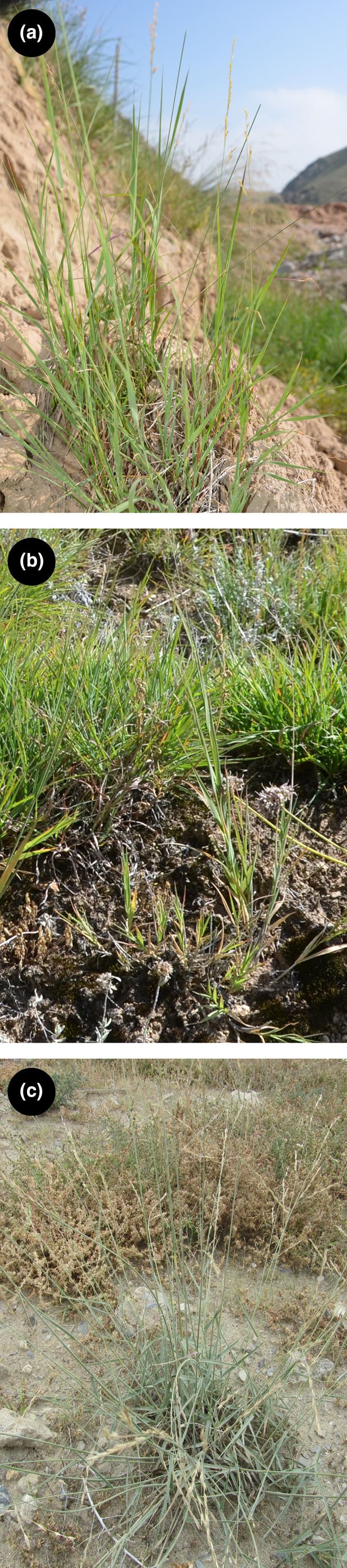
Photographs showing the species of *Orinus* in their habitats: (a) *O. kokonoricus*, (b) *O. intermedius*, and (c) *O*. *thoroldii*

Within the alpine grasslands of the QTP, the dominant vascular plants are three endemic species comprising the entirety of the genus, *Orinus* Hitchcock (Figure 1; Poaceae; Liu et al., 2018; Su, Wu, Li, & Liu, 2015). *Orinus* consists of clonal grasses and was established in 1933 by Hitchcock based on the type species *O*. *arenicola* Hitchc. [=*O*. *thoroldii* (Stapf ex Hemsl.) Bor] collected in the Kashmir region. The genus is sister to *Cleistogenes* Keng in subtribe Orininae P. M. Peterson, Romasch. & Y. Herrera from the QTP (Peterson, Romaschenko, & Arrieta, 2016; Soreng et al., 2017). The species of *Orinus* occur especially in high‐elevation, xeric areas of the QTP. Among the three species, *Orinus thoroldii* is primarily distributed in the western QTP, *O*. *kokonoricus* (K. S. Hao) Tzvelev occurs in the eastern QTP, and *O*. *intermedius* X. Su & J. Quan Liu is native to the southeastern QTP.

*Orinus* is especially characterized by long scaly rhizomes with numerous nodes, which serve as the basis for its clonal reproduction. It also reproduces sexually via seeds borne on sparse panicles within pedicelled and laterally compressed spikelets that have 3‐ to 5‐veined lemmas with short awns (Su, Liu, Wu, Luo, & Liu, 2017). *Orinus thoroldii* is distinguished from *O. kokonoricus* by having pubescent leaf blades and dark brown or purple spikelets with two to six flowers (Su et al., 2017). Leaf blades in *O. kokonoricus* are glabrous and spikelets are yellow or white and bear one to three flowers (Su et al., 2017). In a recent taxonomic revision of the genus, Su et al. (2017) described *O. intermedius*, a new species, as most similar to *O. kokonoricus* but bearing intermediate features between *O. kokonoricus* and *O. thoroldii*, such as having caryopses and stamen of intermediate lengths. Su et al. (2017), Su et al. (2015) recognized *O. intermedius* as distinct on account of its rhizomes bearing sparse small scales compared to *O. kokonoricus* and *O. thoroldii*, which have many larger scales. However, Su et al. (2015) suspected that *O. intermedius* may have a hybrid origin with the other two species as progenitors. Nevertheless, *O. intermedius* appeared more likely to be an incompletely isolated sister of *O*. *kokonoricus* than a hybrid based on a population‐level phylogenetic study comprising chloroplast and nuclear ribosomal internal transcribed spacer (ITS; Liu et al., 2018). At present, the putative hybrid status of *O. intermedius* remains incompletely resolved.

*Orinus* represents an important model for evolution and biodiversity of vascular plants within the grasslands of the QTP for several reasons. As the dominant vascular plant species within the xeric, alpine grasslands, *Orinus* can provide a representative first glimpse into evolutionary diversification and diversity within this threatened habitat type (Ma et al., 2017; Sedlacek et al., 2016; Yang et al., 2004). Moreover, few population genetics studies have targeted clonal species, which may exhibit different patterns of diversification than species that most often reproduce sexually. Finally, *Orinus* possesses an extensive system of roots and rhizomes (Cai, 2004; Su et al., 2015; Su, Yue, & Liu, 2013) that limit soil loss within the wind‐swept alpine grasslands of the QTP (Figure 1; Yang et al., 2004). Thus, the diversity and diversification of the genus can also yield insights into the timing, mechanisms, and ecological consequences of regional desertification (Guo et al., 2002; Han, Fang, & Berger, 2012; see also Liu et al., 2018).

In this report, we investigated diversity and diversification in *Orinus* using analyses of amplified fragment length polymorphism (AFLP) markers. We specifically sought to address the following questions: (a) Are there three distinct species of *Orinus*, and do these exhibit recent or ongoing gene flow? and (b) Does *O. intermedius* have a hybrid origin? Additionally, we used our data to compare patterns of diversity and diversification in *Orinus* to other clonal plants, especially of alpine regions.

## 2 MATERIALS AND METHODS

### 2.1 Taxonomic sampling strategy and obtaining AFLPs

The AFLPs analyzed in this study were previously published in Liu et al. (2018) where they were used in a distance‐based phylogenetic analysis complementary to phylogenetic reconstructions based on chloroplast and nuclear gene sequences. Here, we analyzed the AFLPs for the first time using population genetics methods and applied them to perform the first explicit test of the hybrid origin hypothesis for *O. intermedius*. Below, we describe obtaining the AFLPs, including taxonomic sampling, in brief, and refer to our prior work for greater detail (Liu et al., 2018).

**Figure 2:**
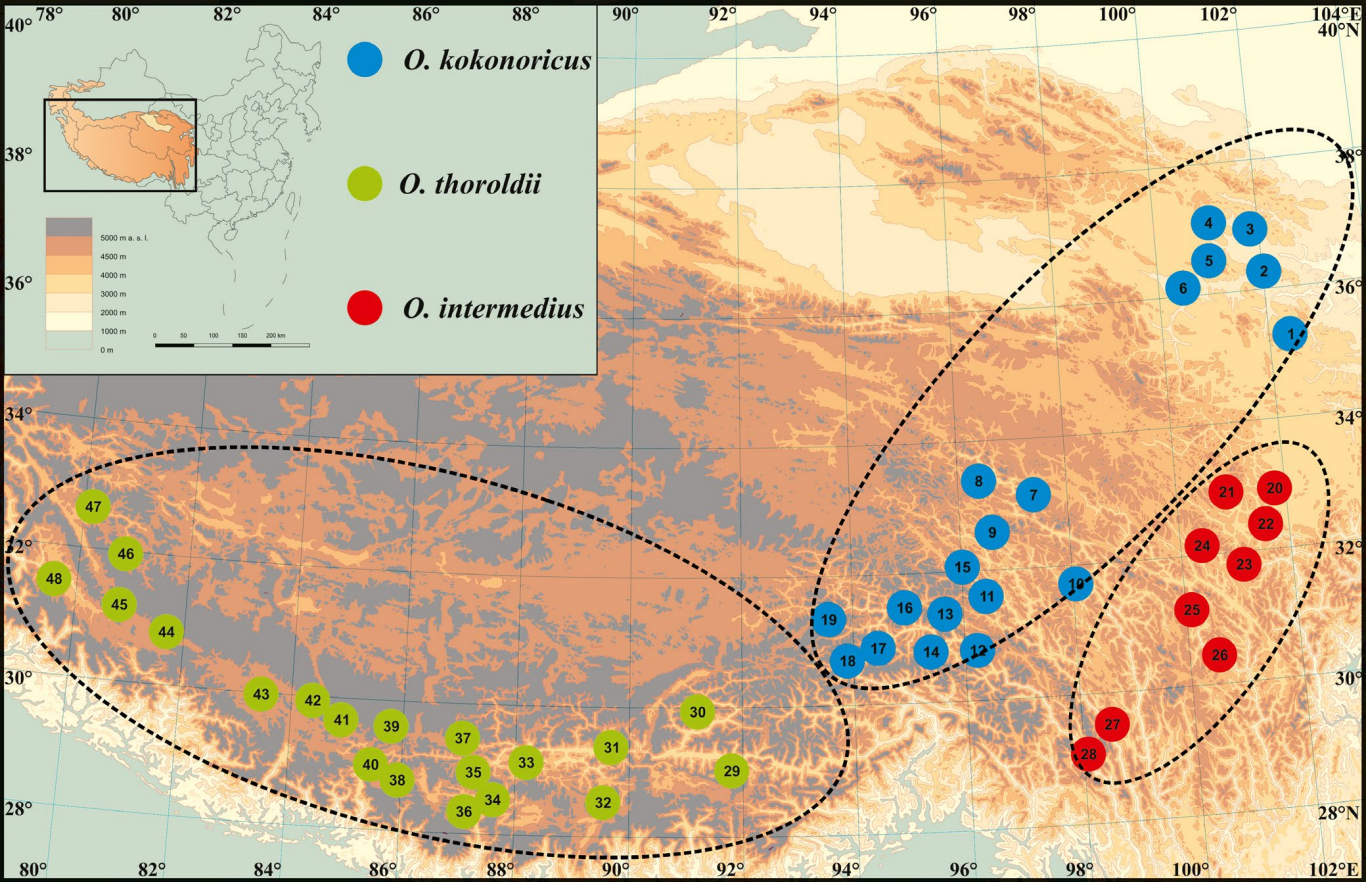
Localities of *O*. *thoroldii* (green), *O*. *kokonoricus* (blue), and *O*. *intermedius* (red) sampled in this study

We sampled a total of 231 individuals of the genus *Orinus* from 48 natural populations from 28°21′51.0 to N and 79°48′9.0 to 102°30′59.7E representing the distributional ranges of the species and including the type localities of each (Figures 1 and 2, Table 1). As species of *Orinus* are dominant within the grasslands of the QTP, the boundaries among populations can be difficult to determine. Thus, we sampled from localities at least 30 km apart to ensure, to the best of our abilities, the genetic independence of the sampling localities except via dispersal of pollen, seeds, or propagules. Per population, we collected fresh leaf blades from three to five vegetative units spaced at least 20 m apart in order to try and sample genetically unique individuals of this clonal species. Our sampling protocol was designed to detect the diversity of genotypes within and among populations covering a vast region, especially to capture rare alleles (e.g., as in Pluess & Stöcklin, 2004), and, notably, our objectives do not include determining the abundance of clonal genotypes within populations at this time. Nevertheless, we regard our within‐population sampling as preliminary and acknowledge that greater depth of sampling will yield deeper insights into some aspects of diversity and diversification in the genus in future studies. We dried the leaf samples in silica gel. For each population, voucher specimens and geolocations are reported in Liu et al. (2018).

**Table 1:** Localities for samples of *Orinus* collected for this study

Population code	Species name	Locality	*N*	Latitude (N)	Longitude (E)	Altitude (m)	Voucher specimens
1	*O*. *kokonoricus*	Xiahe, Gansu	5	35°11′9.2″	102°30′59.7″	3,007	X. Su, 11,295
2		Gonghe, Qinghai	5	36°11′3.0″	101°59′16.9″	2,826	X. Su, 12,040
3		Xining, Qinghai	5	36°37′10.8″	101°44′1.7″	2,547	X. Su, 12,042
4		Haiyan, Qinghai	5	36°50′8.3″	100°50′6.1″	3,305	X. Su, 11,005
5		Gonghe, Qinghai	5	36°6′0.5″	100°24′16.0″	2,998	X. Su, 11,016
6		Gonghe, Qinghai	5	36°2′21.8″	100°18′55.6″	3,072	X. Su, 12,038
7		Yushu, Qinghai	5	32°58′55.6″	97°14′17.6″	3,493	X. Su, 13,095
8		Nangqian, Qinghai	5	32°32′50.6″	96°11′45.2″	4,119	X. Su, 11,075
9		Nangqian, Qinghai	5	32°29′24.4″	96°16′7.5″	3,728	X. Su, 11,080
10		Jiangda, Xizang	5	31°20′20.8″	98°8′2.2″	3,818	X. Su, 12,032
11		Changdu, Xizang	5	31°15′20.0″	97°9′42.4″	3,298	X. Su, 12,025
12		Changdu, Xizang	5	31°29′36.7″	97°12′21.1″	3,354	X. Su, 12,027
13		Dingqing, Xizang	5	31°15′57.4″	95°49′57.0″	3,603	X. Su, 11,152
14		Luolong, Xizang	5	30°46′1.2″	95°34′27.7″	3,762	X. Su, 13,081
15		Leiwuqi, Xizang	4	31°45′12.8″	96°19′51.2″	3,624	X. Su, 13,090
16		Dingqing, Xizang	5	31°36′18.9″	95°6′53.8″	3,786	X. Su, 13,087
17		Bianba, Xizang	5	30°49′19.1″	94°51′30.7″	3,999	X. Su, 13,082
18		Bianba, Xizang	5	30°58′40.3″	94°43′35.3″	3,597	X. Su, 13,083
19		Biru, Xizang	5	31°31′7.8″	93°31′59.7″	3,991	X. Su, 13,085
20	*O*. *intermedius*	Aba, Sichuan	5	32°45′26.7″	102°3′33.8″	3,319	X. Su, 12,003
21		Banma, Qinghai	4	33°1′28.9″	100°41′52.3″	3,852	X. Su, 13,032
22		Aba, Sichuan	4	32°54′45.0″	101°46′59.3″	3,379	X. Su, 11,285
23		Aba, Sichuan	5	32°54′28.2″	101°46′25.5″	3,358	X. Su, 12,001
24		Aba, Sichuan	4	31°46′16.2″	100°58′57.1″	3,478	X. Su, 12,007
25		Luhuo, Sichuan	5	31°38′35.0″	100°17′15.9″	3,534	X. Su, 13,058
26		Daofu, Sichuan	3	30°37′17.7″	101°24′15.5″	3,573	X. Su, 12,008
27		Mangkang, Xizang	5	29°32′28.8″	98°15′18.5″	3,522	X. Su, 13,075
28		Mangkang, Xizang	4	29°32′27.2″	98°15′3.3″	3,507	X. Su, 12,016
29	*O*. *thoroldii*	Zhanang, Xizang	4	29°15′23.9″	91°22′7.1″	3,586	X. Su, 11,195
30		Qushui, Xizang	5	29°29′46.0″	90°56′14.6″	3,617	X. Su, 11,010
31		Rikaze, Xizang	5	29°18′0.4″	89°46′7.3″	3,767	X. Su, 11,018
32		Kangma, Xizang	5	28°33′20.0″	89°41′2.0″	4,412	X. Su, 11,132
33		Lazi, Xizang	5	29°9′28.3″	88°10′16.9″	4,060	X. Su, 11,033
34		Dingjie, Xizang	5	28°21′51.0″	87°45′57.0″	4,324	X. Su, 11,120
35		Dingri, Xizang	5	28°39′34.2″	87°7′45.6″	3,852	X. Su, 11,123
36		Dingri, Xizang	5	28°39′34.2″	87°7′45.6″	3,852	X. Su, 11,119
37		Angren, Xizang	5	29°26′24.0″	86°39′52.6″	4,593	X. Su, 11,034
38		Jilong, Xizang	5	28°46′6.3″	85°32′14.3″	4,614	X. Su, 11,100
39		Shaga, Xizang	4	29°23′31.5″	85°30′57.4″	4,677	X. Su, 11,039
40		Shaga, Xizang	5	29°0′27.0″	85°26′48.8″	4,687	X. Su, 11,078
41		Shaga, Xizang	5	29°30′1.4″	84°33′39.6″	4,578	X. Su, 11,043
42		Zhongba, Xizang	5	29°41′7.9″	84°8′48.1″	4,563	X. Su, 11,044
43		Zhongba, Xizang	5	29°59′45.6″	83°31′43.1″	4,582	X. Su, 11,045
44		Pulan, Xizang	5	30°48′35.8″	81°34′22.5″	4,610	X. Su, 11,049
45		Pulan, Xizang	5	30°21′58.5″	81°9′8.3″	4,260	X. Su, 11,050
46		Pulan, Xizang	5	31°10′42.6″	80°45′26.8″	4,427	X. Su, 11,054
47		Ali, Xizang	5	32°34′17.9″	80°3′10.7″	4,451	X. Su, 11,056
48		Zhada, Xizang	5	31°28′46.0″	79°48′9.0″	4,434	X. Su, 11,070

Abbreviation: *N*, number of individuals sampled for amplified fragment length polymorphism experiments.

**Figure 3:**
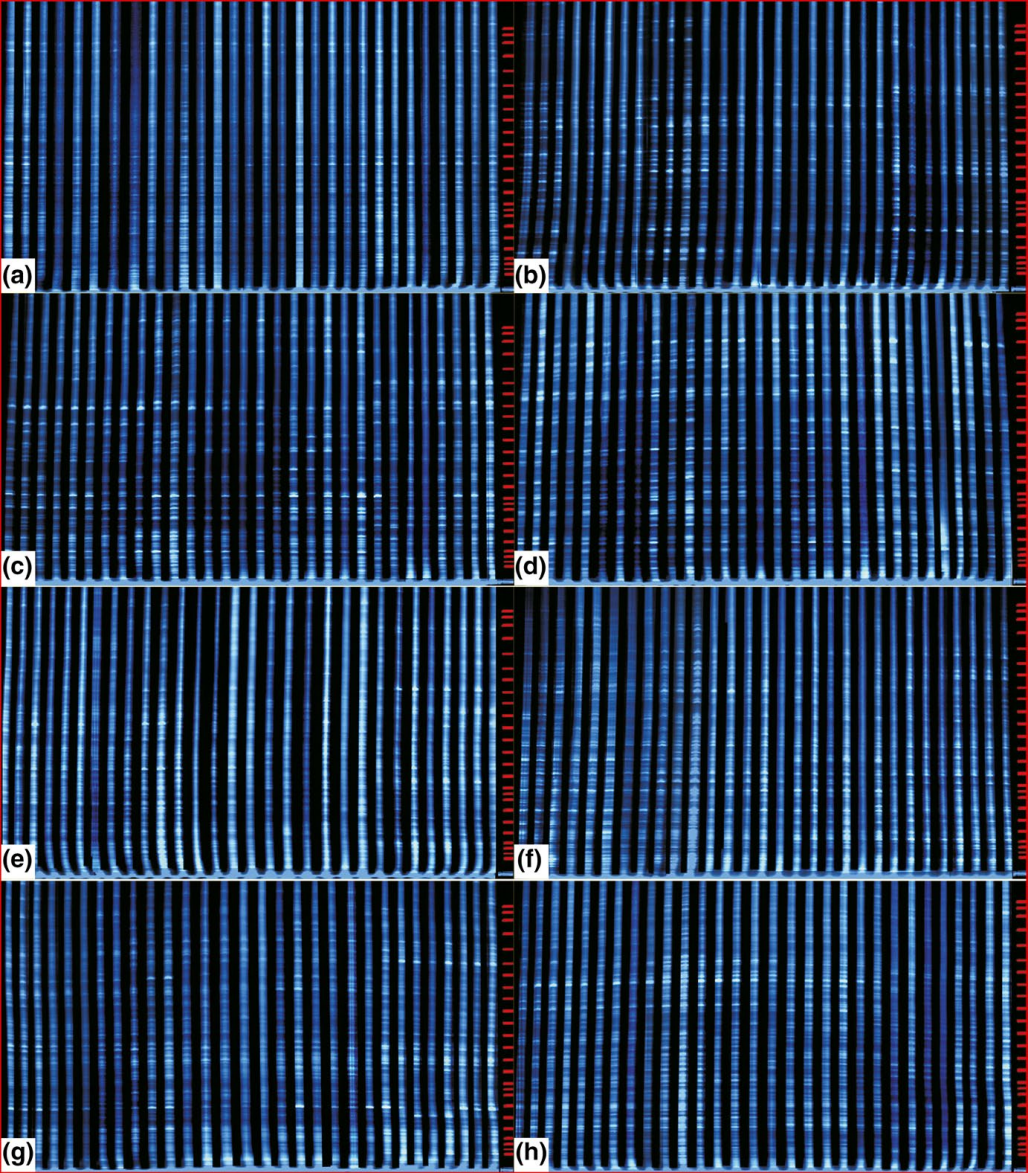
Fluorescently‐labeled AFLPs generated using different primer combinations. (a) P‐GAA/M‐CAA, (b) P‐GAC/M‐CAC, (c) P‐GAC/M‐CTG, (d) P‐GAG/M‐CTA, (e) P‐GAG/M‐CAA, (f) P‐GAG/M‐CAG, (g) P‐GAG/M‐CTG, and (h) P‐GAT/M‐CAG

For the AFLP analyses of all individuals, we performed DNA digestion with DNAs obtained using standard methods (Doyle & Doyle, 1987; see Liu et al., 2018) and the restriction enzymes *Pst*I and *Mse*I (40 U/μl; Beijing Dingguo Biotechnology Co., Ltd). We performed two rounds of PCR on the digestion products comprising preamplification and selective amplification (Table 2). We carried out selective amplification (Zuo, Wen, Ma, & Zhou, 2015) in 25 μl volume of reaction mixture containing of 2.0 μl *Pst*I/*Mse*I primer combinations (GAA/CAA, GAC/CAC, GAC/CAG, GAC/CTA, GAG/CAA, GAG/CAG, GAG/CTG, and GAT/CAG; Table 2). Subsequently, we separated and analyzed the fluorescently‐labeled amplification products on an ABI PRISM 377 DNA Sequencer (Applied Biosystems) using GeneScan ROX‐500 with an internal size standard. We scored the presence or absence of the resulting AFLP products (Figure 3) using GeneScan 3.1 (Applied Biosystems). We imported the scored data into Binthere (Garnhart, 2001) and MG (Zhou & Qian, 2003) to generate a presence/absence, or 0/1 binary, matrix (data available from the Dryad Digital Repository: https://doi.org/10.5061/dryad.403j5s4) for downstream analyses.

**Table 2:** Adapters and primer combination sequences used in this study

Primer	Name	Sequence
Adapters		
P‐L	*Pst* I‐adapter	5′‐CTCGTAGACTGCGTACATGCA‐3′
P‐R	*Pst* I‐adapter	5′‐TGTACGCAGTCTAC‐3′
M‐L	*Mse* I‐adapter	5′‐GACGATGAGTCCTGAG‐3′
M‐R	*Mse* I‐adapter	5′‐TACTCAGGACTCAT‐3′
Preamplification primer		
P01	*Pst* I	5′‐GACTGCGTACATGCAG‐3′
P02	*Mse* I	5′‐GATGAGTCCTGAGTAAC‐3′
Selective amplification primer		
A‐1	*Pst* I‐GAA	5′‐GACTGCGTACATGCAGAA‐3′
	*Mse* I‐CAA	5′‐GATGAGTCCTGAGTAACAA‐3′
B‐2	*Pst* I‐GAC	5′‐GACTGCGTACATGCAGAC‐3′
	*Mse* I‐CAC	5′‐GATGAGTCCTGAGTAACAC‐3′
B‐3	*Pst* I‐GAC	5′‐GACTGCGTACATGCAGAC‐3′
	*Mse* I‐CAG	5′‐GATGAGTCCTGAGTAACAG‐3′
B‐5	*Pst* I‐GAC	5′‐GACTGCGTACATGCAGAC‐3′
	*Mse* I‐CTA	5′‐GATGAGTCCTGAGTAACTA‐3′
C‐1	*Pst* I‐GAG	5′‐GACTGCGTACATGCAGAG‐3′
	*Mse* I‐CAA	5′‐GATGAGTCCTGAGTAACAA‐3′
C‐3	*Pst* I‐GAG	5′‐GACTGCGTACATGCAGAG‐3′
	*Mse* I‐CAG	5′‐GATGAGTCCTGAGTAACAG‐3′
C‐7	*Pst* I‐GAG	5′‐GACTGCGTACATGCAGAG‐3′
	*Mse* I‐CTG	5′‐GATGAGTCCTGAGTAACTG‐3′
D‐3	*Pst* I‐GAT	5′‐GACTGCGTACATGCAGAT‐3′
	*Mse* I‐CAG	5′‐GATGAGTCCTGAGTAACAG‐3′

### 2.2 Genetic diversity and population genetic structure

For each population, we calculated the average standard deviation among markers. Thus, a population with all 1s or 0s for a particular marker would have a standard deviation of zero for the marker, and clonal individuals should have an average deviation of zero. However, clonal individuals may vary in AFLP analyses due to errors in obtaining or processing the data or due to somatic mutations. Thus, we regarded any population with less than 0.05 average deviation as being comprised exclusively of clones, and we sought to exclude these populations from downstream analyses.

We assessed genetic diversity in *Orinus*, including natural breaks potentially corresponding to species, by analyzing binary matrix of AFLP bands. We analyzed the matrix in POPGENE 1.32 (Yeh, Yang, & Boyle, 1999) to calculate the following summary statistics: percentage of polymorphic loci (*PPL*), observed number of alleles (*N*_a_), effective number of alleles (*N*e), expected heterozygosity (*H*_e_; Kimura & Crow, 1964), and Shannon's information index (*I*; Lewontin, 1972). We also analyzed the binary matrix using the NTSYS‐pc 2.l statistical package (Rohlf, 2000). Specifically, in NTSYS, we generated a pairwise similarity matrix with a simple matching coefficient according to the SIMQUAL algorithm. We also used SAHN in NTSYS package to construct a UPGMA tree based on Nei's genetic distance for assessment of relationships among individuals and populations of *Orinus*, and we estimated support for the UPGMA tree using 2000 bootstrap replicates in Winboot software (Yap & Nelson, 1996; see also Liu et al., 2018). We calculated a genetic similarity matrix from the AFLP data according to the method of Nei and Li (1979) and visualized genetic variation among individuals with a principal coordinate analysis (PCoA) performed in GENALEX 6.5 (Peakall & Smouse, 2012). In addition, we constructed a similarity‐based network using the Neighbor‐Net algorithm based on Jaccard's distances within SplitsTree 4.13 (Huson & Bryant, 2006) to further depict relationships among individuals and populations and species based on the AFLP datasets.

We also sought to evaluate the genetic differentiation between and within populations of the three species of *Orinus* using average *F*_ST_, analysis of molecular variance (AMOVA; Excoffier, Smouse, & Quattro, 1992), and a Mantel test. We calculated *F*_ST_ using Arlequin 3.11 (Excoffier, Laval, & Schneider, 2005) and determined significance of the pairwise *F*_ST_ comparisons via permutation tests (*n* = 1,000) with a sequential Bonferroni correction. For the AMOVA, we tested significance with nonparametric permutation using 9,999 replications. We performed Mantel tests on the distance matrix of Jaccard's coefficients calculated in GENALEX 6.5 (Peakall & Smouse, 2012) in order to detect the correlations between genetic distances generated from each of the AFLP primer pairs, and geographic distances of populations derived from geographic coordinates using AFLP datasets (Ehrich, 2006). For the Mantel tests, we computed correlation coefficients and assessed the significance with 1,000 permutations.

We conducted a Bayesian analysis of the population structure in *Orinus* using STRUCTURE 2.3 (Falush, Stephens, & Pritchard, 2007; Hubisz, Falush, Stephens, & Pritchard, 2009; Pritchard, Stephens, & Donnelly, 2000) to determine whether the structure was consistent with species boundaries and to infer the relative amounts of gene flow between each species. We performed the analyses using an admixture model with independent allele frequencies for 10 independent runs for the number of clusters (*K*) ranging from 1 to 10. We applied 1 × 10^6^ Markov chain Monte Carlo repetitions with a burn‐in rate of 25%. We summarized the outputs of all runs with the Web‐based software Structure Harvester (Earl & von, 2012), and we calculated the average similarity coefficients among runs for each *K*. We determined the optimal *K* using two methods: the point of diminishing returns for adding additional *K* (i.e., elbow method) and the value representing the greatest change from the previous value (i.e., Δ*K*; Evanno, Regnaut, & Goudet, 2005; Pritchard et al., 2000).

### 2.3 Testing AFLP data against explicit genetic models of hybridization

We tested the hybrid status of *O. intermedius* using the Bayesian implementation in NewHybrids (Anderson & Thompson, 2002) version 2.0+ Developmental (https://github.com/eriqande/newhybrids). Specifically, we tested the 231 sampled individuals for their compatibility with five genetic models: that each is genetically (a) *O. kokonoricus*, (b) *O. thoroldii,* (c) a true hybrid of *O. kokonoricus* and *O. thoroldii*, (d) a hybrid of *O. kokonoricus* and *O. thoroldii* backcrossed with *O. kokonoricus*, and (e) a hybrid of *O. kokonoricus* and *O. thoroldii* backcrossed with *O. thoroldii*. These models cannot explicitly test the possibility that *O. intermedius* is an independent species not derived from hybrid origins. However, *O. intermedius* individuals should be resolved under model 1 or 2 if the species is not a hybrid but, instead, shared a common ancestor with either *O. kokonoricus* or *O. thoroldii* that does not include the other species. Additionally, models 4 and 5 cannot be differentiated from low levels of introgression that may occur among species that are differentiating in allopatry, but we interpret these results within the context of our other statistical analyses. For individuals within populations, we averaged the posterior probabilities of compatibility with each model. Thus, our results represent the average posterior probability for the best genetic model for each population. We also present the results for each individual in Appendix A.

## 3 RESULTS

### 3.1 Genetic diversity

Among 64 pairs of *EcoR*I/*Mse*I primer combinations, we successfully obtained eight pairs of selective AFLP primers that could amplify fragments with good coverage in the 231 individuals representing 48 populations of the three species of *Orinus* (Table 1, Figure 3). For the eight primer pairs, all summary and genetic statistics for the primer pairs are presented in Table 3. The eight pairs produced a total of 1,324 unambiguous and repetitious AFLP amplification bands across all the samples from *O*. *thoroldii* and *O*. *kokonoricus*, and 1,261 in *O*. *intermedius*. The total number of AFLP amplification bands for each primer pair ranged from 154 (P‐GAC/M‐CAC) to 185 (P‐GAT/M‐CAG) with an average of 166, 150 (P‐GAA/M‐CAA) to 179 (P‐GAC/M‐CAC) with an average of 166, and 143 (P‐GAA/M‐CAA) to 171 (P‐GAG/M‐CAA) with an average of 158. Among the AFLP amplification bands, 1,313 (99.17%) were polymorphic in *O*. *thoroldii*, 1,315 (99.32%) in *O*. *kokonoricus*, and 1,242 (98.49%) in *O*. *intermedius*. The total number of polymorphic bands for each primer pair varied from 152 (P‐GAC/M‐CAC) to 185 (P‐GAT/M‐CAG) with an average of 164, 150 (P‐GAA/M‐CAA) to 179 (P‐GAC/M‐CAC) with an average of 164, and 142 (P‐GAA/M‐CAA) to 171 (P‐GAG/M‐CAA) with an average of 155. Each primer pair yielded rich and clear patterns among the three species of *Orinus*. The allele size of *O*. *thoroldii* and *O*. *intermedius* ranged from 70 to 500 bp, while that of *O*. *kokonoricus* ranged from 60 to 500 bp. In addition, the percentage polymorphism of each species of *Orinus* varied from 98.11% to 100% with an average of 99.15% in *O*. *thoroldii*, 95.78% to 100% with an average of 99.32% in *O*. *kokonoricus*, and 96.27% to 100% with an average of 98.26% in *O*. *intermedius* among the primer pairs.

**Table 3:** Summary Statistics for eight amplified fragment length polymorphism selective primer combinations in the present study

Species name	Selective nuclear	Polymorphism bands	Amplification bands	PPL (%)	Size range (bp)	*N*_a_	*N*_e_	*H*_e_	*I*
*O. thoroldii*	P‐GAA/M‐CAA	161	164	98.17	70–500	1.98	1.33	0.20	0.32
	P‐GAC/M‐CAC	152	154	98.70	70–500	1.99	1.33	0.20	0.31
	P‐GAC/M‐CAG	167	170	98.24	70–500	1.98	1.27	0.17	0.27
	P‐GAC/M‐CTA	156	159	98.11	71–498	1.98	1.30	0.19	0.30
	P‐GAG/M‐CAA	174	174	100	70–499	2.00	1.30	0.19	0.31
	P‐GAG/M‐CAG	159	159	100	70–500	2.00	1.34	0.21	0.33
	P‐GAG/M‐CTG	159	159	100	70–494	2.00	1.33	0.21	0.33
	P‐GAT/M‐CAG	185	185	100	71–498	2.00	1.33	0.21	0.34
	Total	1,313	1,324	99.17	560–3,988	–	–	–	–
	Mean	164	166	99.15	70–499	1.99	1.32	0.20	0.31
*O. kokonoricus*	P‐GAA/M‐CAA	150	150	100	70–500	2.00	1.37	0.23	0.35
	P‐GAC/M‐CAC	179	179	100	70–498	2.00	1.29	0.19	0.31
	P‐GAC/M‐CAG	159	166	95.78	71–498	1.96	1.29	0.18	0.29
	P‐GAC/M‐CTA	161	163	98.77	70–499	1.99	1.32	0.20	0.31
	P‐GAG/M‐CAA	170	170	100	70–499	2.00	1.33	0.20	0.33
	P‐GAG/M‐CAG	164	164	100	60–497	2.00	1.32	0.20	0.32
	P‐GAG/M‐CTG	174	174	100	70–493	2.00	1.31	0.20	0.37
	P‐GAT/M‐CAG	158	158	100	71–500	2.00	1.34	0.21	0.33
	Total	1,315	1,324	99.32	551–3,984	–	–	–	–
	Average	164	166	99.32	69–498	1.99	1.32	0.20	0.32
*O. intermedius*	P‐GAA/M‐CAA	142	143	99.30	70–499	1.99	1.38	0.23	0.37
	P‐GAC/M‐CAC	163	163	100	71–498	2.00	1.33	0.21	0.34
	P‐GAC/M‐CAG	155	161	96.27	70–499	1.96	1.29	0.19	0.30
	P‐GAC/M‐CTA	152	154	98.70	70–500	1.99	1.36	0.22	0.35
	P‐GAG/M‐CAA	171	171	98.28	70–489	1.98	1.31	0.20	0.32
	P‐GAG/M‐CAG	142	146	97.26	70–490	1.97	1.34	0.21	0.33
	P‐GAG/M‐CTG	152	155	98.06	70–498	1.98	1.31	0.20	0.33
	P‐GAT/M‐CAG	165	168	98.21	71–497	1.98	1.32	0.21	0.33
	Total	1,242	1,261	98.49	562–3,969	–	–	–	–
	Average	155	158	98.26	70–496	1.98	1.33	0.21	0.33

Note

PPL, percentage of polymorphic loci; *N*_a_, observed number of alleles; *N*_e_, effective number of alleles; *H*_e_, expected heterozygosity; *I*, Shannon's information index.

The mean *N*_a_ of *O*. *thoroldii* was 1.99, which varied from 1.98 to 2.00, while the mean *N*_e_ and *H*_e_ varied from 1.27 to 1.34 with a mean value of 1.32 and from 0.17 to 0.21 with the mean value of 0.20, respectively. The mean value of *I* was 0.31 and ranged from 0.27 to 0.34. For *Orinus kokonoricus*, the *N*_a_, *N*_e_, *H*_e_, and *I* ranged, respectively, from 1.96 to 2.00, 1.29 to 1.37, 0.18 to 0.23, and 0.29 to 0.35. The mean values were 1.99, 1.32, 0.20, and 0.32, also respectively. Similarly, the mean values of *N*_a_, *N*_e_, *H*_e_, and *I* for *O*. *intermedius* were 1.98, 1.33, 0.21, and 0.33, and the variation of these ranged from 1.96 to 2.00, 1.29 to 1.38, 0.19 to 0.23, and 0.30 to 0.37, all respectively. All measures revealed high levels of genetic diversity among the three species of *Orinus*. In particular, *O*. *intermedius* showed the highest level of genetic diversity among the three species according to Shannon's information index (*I* = 0.33).

### 3.2 Population genetic structure

Analysis of molecular variance (AMOVA) based on AFLP datasets and inbreeding coefficients (*F*_ST_; Table 4) indicated significant interspecific genetic differentiation across the natural distribution of the three species of *Orinus* (*F*_ST_ = 0.19, *p* < 0.01). Variation within populations represented 56.65% of the total genetic variation, while variation among species comprised 30.04%, and variation among populations within each species was 13.31%. Among the three species of *Orinus*, the genetic variation between *O*. *thoroldii* and *O*. *kokonoricus* was the highest (*F*_ST_ = 0.46, *p* < 0.01), with 33.67% of the variation between species and 54.34% within populations. For *O*. *thoroldii* and *O*. *intermedius*, the genetic variation was also high (*F*_ST_ = 0.44, *p* < 0.01), and 31.21% of the total variation was interspecific while 55.91% was within populations. Genetic variation between *O*. *kokonoricus* and *O*. *intermedius* was lower, at 18.00% with a corresponding average *F*_ST_ value of 0.35, while 65.04% of the variation was within populations. In all species, intrapopulational genetic variation was much higher than interpopulational.

**Table 4:** Results of analyses of molecular variance (AMOVAs) based on amplified fragment length polymorphism markers for the three species of *Orinus*

Grouping regions	Source of variation	*df*	SS	VC	Percent variation (%)	Fixation index
*O. thoroldii*	Among populations	19	4,692.26	25.25	16.86	*F*_ST_ = 0.17*
	Within populations	77	9,589.10	124.53	83.14	
*O. kokonoricus*	Among populations	18	4,905.54	29.99	19.45	*F*_ST_ = 0.20*
	Within populations	75	9,313.95	124.19	80.55	
*O. intermedius*	Among populations	8	2,340.80	38.79	23.68	*F*_ST_ = 0.24*
	Within populations	30	3,749.92	124.80	76.32	
*O. thoroldii* and *O. kokonoricus*	Among species	1	7,664.98	77.17	33.67	*F*_ST_ = 0.46*
	Among populations within species	37	9,609.03	27.46	11.98	*F*_SC_ = 0.18*
	Within populations	153	19,053.35	124.53	54.34	*F*_CT_ = 0.34*
*O. thoroldii* and *O. intermedius*	Among species	1	4,147.07	69.73	31.21	*F*_ST_ = 0.44*
	Among populations within species	27	7,044.29	28.77	12.88	*F*_SC_ = 0.19*
	Within populations	108	13,489.32	124.90	55.91	*F*_CT_ = 0.31*
*O. kokonoricus* and *O. intermedius*	Among species	1	2,171.07	34.42	18.00	*F*_ST_ = 0.35*
	Among populations within species	26	7,246.34	32.45	16.97	*F*_SC_ = 0.21*
	Within populations	105	13,063.87	124.42	65.04	*F*_CT_ = 0.18*
Total	Among species	2	10,080.42	66.08	30.04	*F*_ST_ = 0.19*
	Among populations within species	45	11,949.83	29.27	13.31	*F*_SC_ = 0.43*
	Within populations	183	22,803.27	124.61	56.65	*F*_CT_ = 0.30*

Note

*df*, degrees of freedom; SS, sum of squares; VC, variance components; *F*_ST_, variance among populations; *F*_SC_, variance among populations within species; *F*_CT_, variance among groups relative to total variance. Significant level:

* *p* < 0.01.

**Figure 4:**
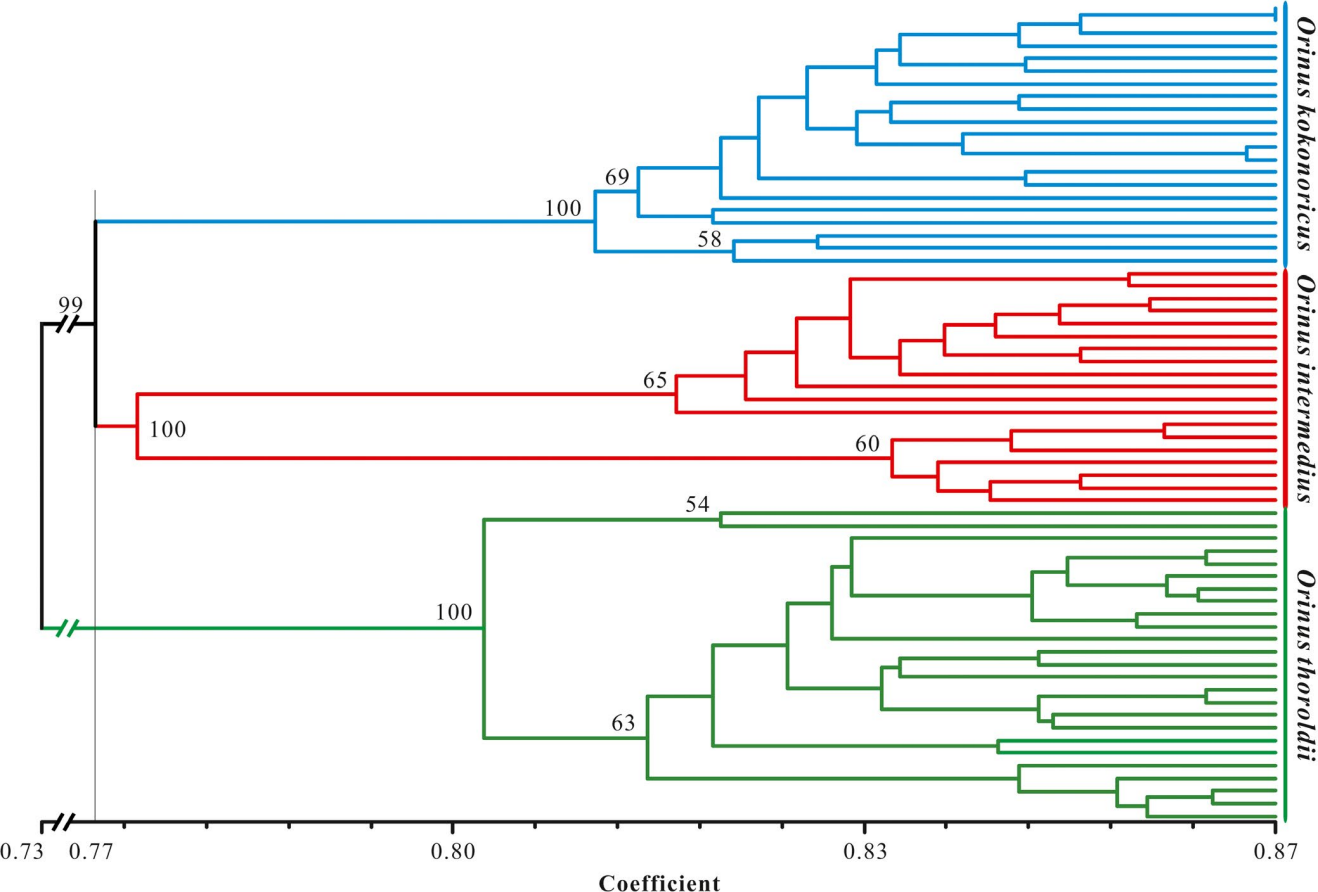
Dendrogram of the three species of *Orinus* generated by unweighted pair group method analysis (UPGMA) cluster analysis from the similarity matrix obtained using amplified fragment length polymorphism genetic distance

**Figure 5:**
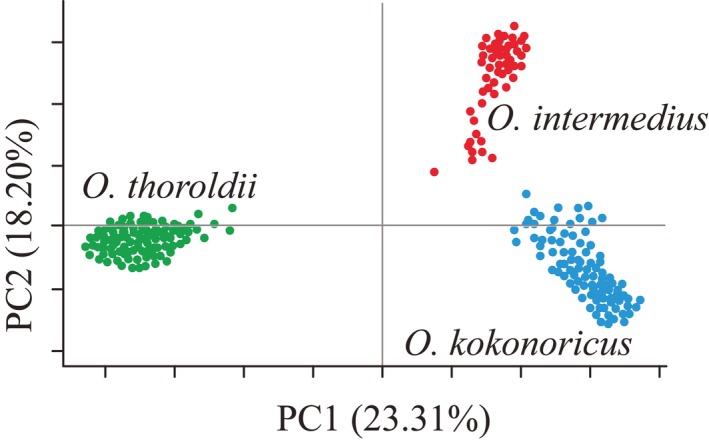
A two‐dimensional plot of the principal coordinate analysis (PCoA)‐based variation of amplified fragment length polymorphism markers within the three species of *Orinus*. Tick marks on axes are in increments of 1.0, and 0.0 on each axis is indicated by a gray line

**Figure 6:**
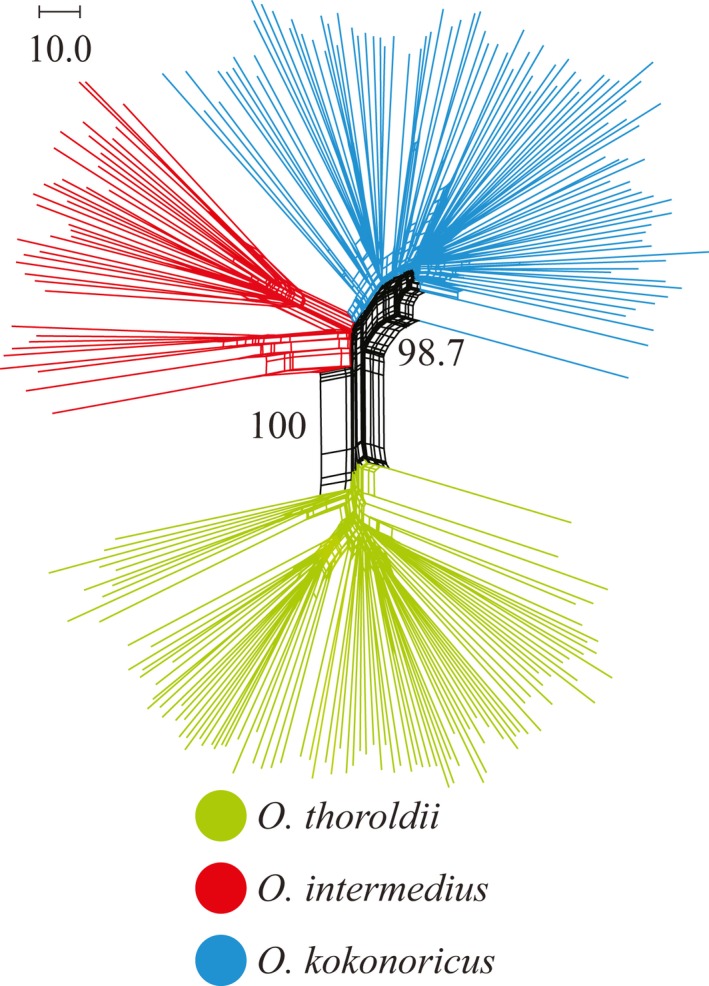
Neighbor‐Net split network of the three species of *Orinus* based on amplified fragment length polymorphism data using Jaccard's distances. Lines of green, blue and red represent *O*. *thoroldii*, *O*. *kokonoricus*, and *O*. *intermedius*, respectively

**Figure 7:**
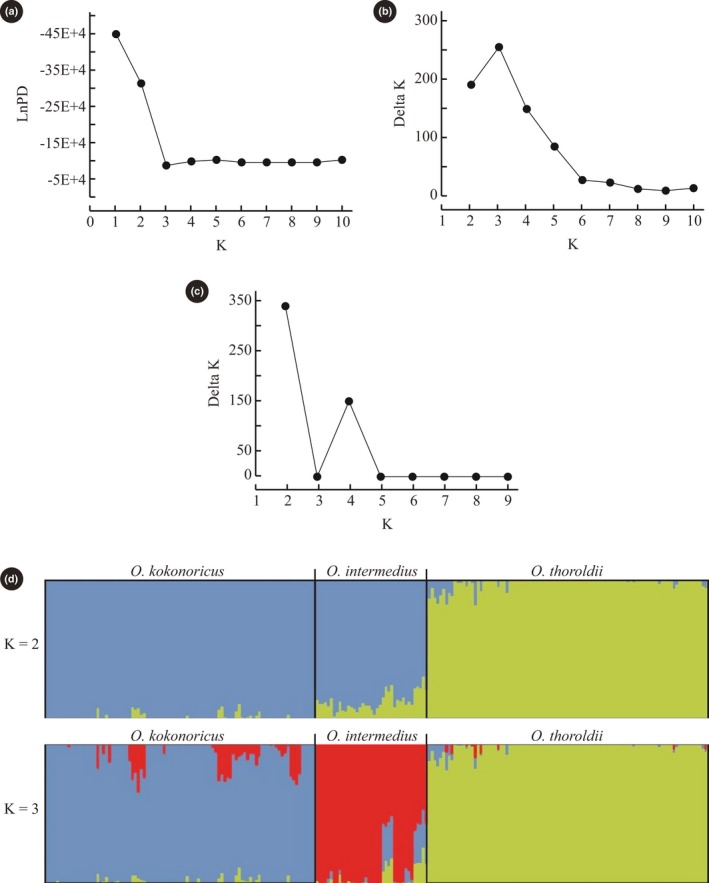
Results of the Bayesian clustering analysis in STRUCTURE of the 231 individuals representing three species of *Orinus*. (a) The probability of the data Ln *P*(*D*) (±*SD*) against the number of *K* cluster, and increase of Ln *P*(*D*) given *K*, calculated as (Ln *P*(*D*)*k*−Ln *P*(*D*)*k*−1). (b) *△K* values from the mean log‐likelihood probabilities from STRUCTURE runs where inferred cluster (*K*) ranged from one to ten. (c) Bayesian inference of the number of clusters (*K*) for the three species of *Orinus*. (d) Estimated genetic clustering for *K* = 2 and 3, where unique colors correspond to assignment to different clusters

The UPGMA tree indicated that the 231 individuals from 48 populations of *Orinus* comprised three clades (Figure 4) corresponding to three geographically clustered groups of populations within the QTP and consistent with species identities. Thus, the UPGMA tree revealed geographic structure within the genus *Orinus* with three independent clades consisting of *O*. *thoroldii*, *O*. *kokonoricus*, and *O*. *intermedius*, which is sister to *O. kokonoricus* (Figure 4). Similarly, the Mantel tests agreed that geography is positively correlated with genetic divergence (*r* = 0.05, *p* < 0.05). Discrete cluster corresponding to species and geography was supported by the principal coordinate analysis (PCoA; Figure 5). The first two axes in the PCoA plot explained 23.31% and 18.20% of variation, respectively (data not shown). The PCoA axis 1 separated *O. thoroldii* from the other two species, while axis 2 yielded greater separation for *O. intermedius*. Additionally, the split network revealed three splits, corresponding to the three species of *Orinus* (Figure 6). According to STRUCTURE (Figure 7), the optimum *K* value was *K* = 3 and the highest peak of Δ*K* values also appeared at *K* = 3. The three clusters predicted by the STRUCTURE analysis corresponded to the three recognized species of *Orinus* (Figure 7d). In a separate analysis with *K* = 2, *O*. *kokonoricus* and *O*. *intermedius* were clustered together (Figure 7d).

### 3.3 Results of genetic models of hybridization

**Figure 8:**
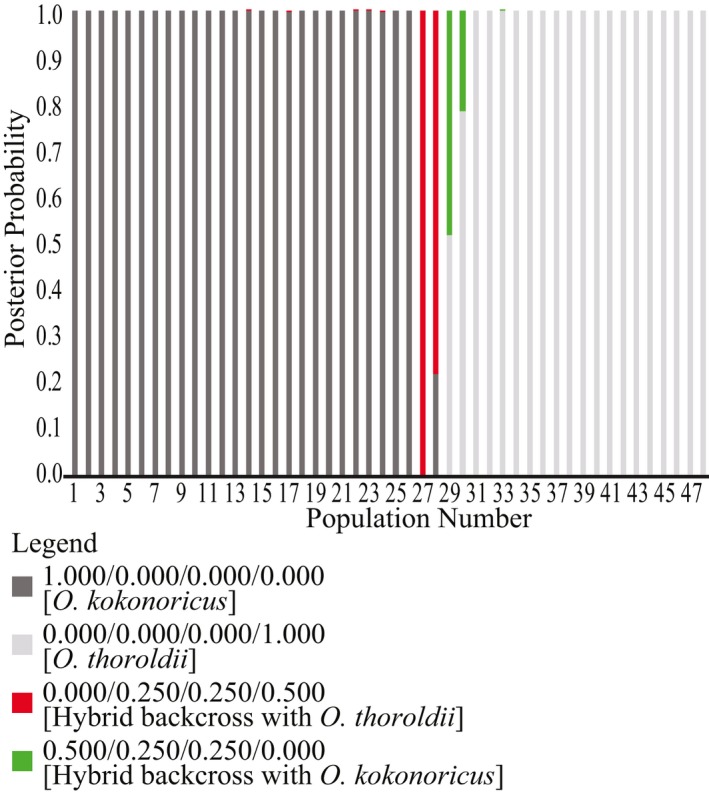
Results of testing explicit genetic models in NewHybrids. Posterior probability of models shown on the *y*‐axis, averaged for populations shown on the *x*‐axis. We tested five models, but the true hybrid model is not shown, because it received 0.0 pp for all individuals and, thus, all populations. Bars represent populations 1‐48 consecutively

The results of NewHybrids show that models of a single genetic origin are best suited to most populations (Figure 8; Appendix A). All populations of *O. kokonoricus* correspond to *O. kokonoricus* origins with posterior probability (pp) of 1.0 except populations 14 and 17 (Figure 2), in which one individual each (Appendix A) showed trivial pp (<0.01) support for hybrid backcrossing into *O. kokonoricus*. Most populations of *O. thoroldii* had 1.0 pp of having a genetic origin of solely *O. thoroldii* stock. For population 33 of *O. thoroldii*, one individual showed a trivial pp of representing a hybrid with backcrossing into *O. thoroldii*. In contrast, populations 29 and 30 had nontrivial pp for representing hybrid backcrosses to *O. thoroldii* (0.48 and 0.21, respectively). Nevertheless, these support values were lower than for an origin from exclusively *O. thoroldii* genetic stock. Among populations of *O. intermedius*, four populations (20, 21, 25, and 26) showed 1.0 pp for exclusive origins from *O. kokonoricus* stock, while three populations (22, 23, and 24) showed high pp for *O. kokonoricus* origins and trivial pp for representing hybrid backcrosses with *O. kokonoricus*. Notably, two populations of *O. intermedius*, 27 and 28, had 1.0 pp and 0.77 pp, respectively, for representing hybrid backcrosses with *O. kokonoricus*.

## 4 DISCUSSION

### 4.1 The hybrid origin of *Orinus intermedius*

In our prior work, we have hypothesized that *Orinus intermedius* (Su et al., 2017) may be either a hybrid of *O. kokonoricus* and *O. thoroldii* or an incompletely diverged sister of *O. kokonoricus*. Here, our AFLP data are consistent with most populations of *O. kokonoricus* and *O. intermedius* sharing a common ancestor, and, thus, a common genetic stock, that is not shared with *O. thoroldii*. Therefore, our data do not support a hybrid origin for *O. intermedius*. However, we observed that two populations of *O. intermedius* are consistent with a backcrossing model. However, this likely represents a level of introgression occurring contemporaneously with speciation processes, rather than backcrossing, as we did not detect any true hybrid individuals or populations.

### 4.2 Species limits in *Orinus* and introgression

Previously, we suggested that *Orinus* represents three species and noted that genetic isolation among all species of *Orinus* is nearly, but not entirely, complete (Liu et al., 2018; Su et al., 2017, 2015). The present study is congruent with our prior work in showing that the three species of *Orinus* are largely distinct, especially according to the UPGMA (Figure 4), STRUCTURE (Figure 7), AMOVA (Table 4), and SplitsTree (Figure 6). However, some gene flow does continue to occur among all species based on the results of these same analyses and may also explain the nonzero probabilities of backcrosses within some populations of *O. kokonoricus* and *O. thoroldii* according to NewHybrids (Figure 8; Appendix A).

Gene flow between *O. intermedius* and *O. kokonoricus* may enable them to maintain the higher levels of genetic diversity that we detected, compared with *O. thoroldii*, which is more genetically isolated. In contrast to our results, it is relatively common that more widely spread species, such as *O. thoroldii*, maintain greater genetic diversity than more geographically restricted species (Hamrick & Godt, 1989; Karron, 1987; Xue, Wang, Korpelainen, & Li, 2005), such as *O. intermedius* and *O. kokonoricus*. High diversity within *O*. *intermedius* and *O*. *kokonoricus* is likely due to their ongoing speciation (Liu et al., 2018), in which barriers to gene flow remain incomplete. Relatedly, due to earlier divergence time of *O. thoroldii*, it may have had more time in isolation to undergo some degree of genetic drift. *Orinus thoroldii* is not only more genetically distant from its congeners (Figure 5), but also more geographically distant. Thus, genetic differentiation in *Orinus* may be mediated by reduced gene flow over greater geographic distances as is consistent with an allopatric mode of speciation as has been observed in other plant species of the QTP (Ge, Zhang, Yuan, Hao, & Chiang, 2005; Hu et al., 2016; Liu, Wang, Geng, et al., 2006; Zhang, Chiang, George, Liu, & Abbott, 2005).

### 4.3 Population and species diversification history in *Orinus* compared to other clonal species

Many clonal species exhibit a common pattern of diversity, which is low or intermediate within populations and very high among them (Ellstrand & Roose, 1987; Li & Ge, 2001). This pattern has been documented in other clonal grasses, such as *Psammochloa villosa* Hitchc. (Poaceae; Li & Ge, 2001; Yu, Dong, & Krüsi, 2004). Overall, for clonal species, this pattern suggests that interpopulation movement of propagules is rare and that diversity within populations may be largely explained by the founder genotypes and, in some cases, outcrossing among genotypes (e.g., *Carex curvula*, *Dryas octopetala* L., *Salix herbacea* L., and *Vaccinium uliginosum* L.; de Witte, Armbruster, Gielly, Taberlet, & Stöcklin, 2012).

*Orinus* differs from other clonal plants by showing the highest diversity within populations and limited diversity among them, including populations within and among species. This is an uncommon pattern of diversity for clonal species, but one which has been previously observed (Pluess & Stöcklin, 2004). In particular, *Geum reptans* L. (Rosaceae), a clonal alpine species of the Swiss Alps, exhibits this pattern of diversity probably due to ongoing gene flow, despite geographic isolation of populations on sky islands (Pluess & Stöcklin, 2004; see also sky islands in Hughes & Atchison, 2015; Körner, 2004). In *Orinus*, gene flow is unlikely to account for this pattern of diversity, especially among species, because the species are, overall, distinct, and because the species probably experience limited gene flow by rare dispersals of rhizome sections and occasional pollen movement by wind, water, and animal visitors. Within *Orinus*, there is no obvious mechanism for seed dispersal. Therefore, alternatively to ongoing, regular gene flow, recency of isolation of species and populations of *Orinus* within the QTP may explain the limited genetic diversity at the interspecific and interpopulational levels, respectively (e.g., as in Cruickshank & Hahn, 2014). Indeed, *Orinus* may have begun diversifying within the QTP during the latter part of the Pliocene (2.85 million years ago; Liu et al., 2018), which represents the end of a global period of evolution of modern alpine species (Hughes & Atchison, 2015). This alternative also requires that the original populations possessed high genetic diversity that has been preserved, at least partially, to present times. High diversity within ancestral populations often results from isolation by vicariance, rather than dispersal, events (Mayr, 1942; see also Harris, Ickert‐Bond, & Rodríguez, 2018; Kropf, Comes, & Kadereit, 2006). Vicariance within the QTP is often invoked to explain commonly observed patterns in the diversification of plant populations or species (e.g., Yang, Li, Ding, & Wang, 2008), especially the divergence of western lineages, such as *O. thoroldii*, from eastern ones, such as *O. kokonoricus* and *O. intermedius*. Moreover, vicariance in the region may be attributed to either the topographic or climatic effects of recent geomorphism (Jia, Liu, Wang, Zhou, & Liu, 2011; Liu et al., 2013; Wen et al., 2014; Yang et al., 2008), and topology may be a better explanation for divergence in the case of *Orinus*, because the ecological niches of species are similar (Su et al., 2015). Overall, the pattern of genetic diversity within *Orinus* could eventually come to resemble patterns overserved for other clonal species (Ellstrand & Roose, 1987; Li & Ge, 2001) given sufficient evolutionary time. However, as a caveat of the present study, we also cannot rule out that our limited sampling within populations accounts for some parts of the patterns in diversity that we observed, and expanded sampling is needed in the future.

## CONFLICT OF INTEREST

None declared.

## AUTHOR CONTRIBUTIONS

XS conceived and designed the study. XS, YL, and QG performed the laboratory work. YL, AJ‐H, QG, XS, and ZR contributed to performing data analyses, interpreting results, and writing the manuscript. All authors approved the manuscript as written.

## Data Availability

All data are provided within the text, tables, appendix, and figures, except for the binary scoring of AFLP bands, which we have submitted to the Dryad Digital Repository (https://doi.org/10.5061/dryad.403j5s4).

## APPENDIX A

**Table A1:** Posterior probabilities for five genetic models tested in NewHybrids for individual samples of species of *Orinus*

Putative Species	Population Number (Figure 2)	1.000/0.000/0.000/0.000 (*O. thoroldii*)	0.000/0.000/0.000/1.000 (*O. kokonoricus*)	0.000/0.500/0.500/0.000 (true hybrid)	0.500/0.250/0.250/0.000 (backcross into *O. thoroldii*)	0.000/0.250/0.250/0.500 (backcross into *O. kokonoricus*)
*O. kokonoricus*	4	0	1	0	0	0
*O. kokonoricus*	4	0	1	0	0	0
*O. kokonoricus*	4	0	1	0	0	0
*O. kokonoricus*	4	0	1	0	0	0
*O. kokonoricus*	4	0	1	0	0	0
*O. kokonoricus*	5	0	1	0	0	0
*O. kokonoricus*	5	0	1	0	0	0
*O. kokonoricus*	5	0	1	0	0	0
*O. kokonoricus*	5	0	1	0	0	0
*O. kokonoricus*	5	0	1	0	0	0
*O. kokonoricus*	8	0	1	0	0	0
*O. kokonoricus*	8	0	1	0	0	0
*O. kokonoricus*	8	0	1	0	0	0
*O. kokonoricus*	8	0	1	0	0	0
*O. kokonoricus*	8	0	1	0	0	0
*O. kokonoricus*	9	0	1	0	0	0
*O. kokonoricus*	9	0	1	0	0	0
*O. kokonoricus*	9	0	1	0	0	0
*O. kokonoricus*	9	0	1	0	0	0
*O. kokonoricus*	9	0	1	0	0	0
*O. kokonoricus*	13	0	1	0	0	0
*O. kokonoricus*	13	0	1	0	0	0
*O. kokonoricus*	13	0	1	0	0	0
*O. kokonoricus*	13	0	1	0	0	0
*O. kokonoricus*	13	0	1	0	0	0
*O. kokonoricus*	1	0	1	0	0	0
*O. kokonoricus*	1	0	1	0	0	0
*O. kokonoricus*	1	0	1	0	0	0
*O. kokonoricus*	1	0	1	0	0	0
*O. kokonoricus*	1	0	1	0	0	0
*O. kokonoricus*	12	0	1	0	0	0
*O. kokonoricus*	12	0	1	0	0	0
*O. kokonoricus*	12	0	1	0	0	0
*O. kokonoricus*	12	0	1	0	0	0
*O. kokonoricus*	12	0	1	0	0	0
*O. kokonoricus*	11	0	1	0	0	0
*O. kokonoricus*	11	0	1	0	0	0
*O. kokonoricus*	11	0	1	0	0	0
*O. kokonoricus*	11	0	1	0	0	0
*O. kokonoricus*	11	0	1	0	0	0
*O. kokonoricus*	10	0	1	0	0	0
*O. kokonoricus*	10	0	1	0	0	0
*O. kokonoricus*	10	0	1	0	0	0
*O. kokonoricus*	10	0	1	0	0	0
*O. kokonoricus*	10	0	1	0	0	0
*O. kokonoricus*	6	0	1	0	0	0
*O. kokonoricus*	6	0	1	0	0	0
*O. kokonoricus*	6	0	1	0	0	0
*O. kokonoricus*	6	0	1	0	0	0
*O. kokonoricus*	6	0	1	0	0	0
*O. kokonoricus*	2	0	1	0	0	0
*O. kokonoricus*	2	0	1	0	0	0
*O. kokonoricus*	2	0	1	0	0	0
*O. kokonoricus*	2	0	1	0	0	0
*O. kokonoricus*	2	0	1	0	0	0
*O. kokonoricus*	3	0	1	0	0	0
*O. kokonoricus*	3	0	1	0	0	0
*O. kokonoricus*	3	0	1	0	0	0
*O. kokonoricus*	3	0	1	0	0	0
*O. kokonoricus*	3	0	1	0	0	0
*O. kokonoricus*	14	0	1	0	0	0
*O. kokonoricus*	14	0	0.99999	0	0	0.00001
*O. kokonoricus*	14	0	1	0	0	0
*O. kokonoricus*	14	0	1	0	0	0
*O. kokonoricus*	14	0	1	0	0	0
*O. kokonoricus*	17	0	1	0	0	0
*O. kokonoricus*	17	0	1	0	0	0
*O. kokonoricus*	17	0	0.99735	0	0	0.00265
*O. kokonoricus*	17	0	1	0	0	0
*O. kokonoricus*	17	0	1	0	0	0
*O. kokonoricus*	18	0	1	0	0	0
*O. kokonoricus*	18	0	1	0	0	0
*O. kokonoricus*	18	0	1	0	0	0
*O. kokonoricus*	18	0	1	0	0	0
*O. kokonoricus*	18	0	1	0	0	0
*O. kokonoricus*	19	0	1	0	0	0
*O. kokonoricus*	19	0	1	0	0	0
*O. kokonoricus*	19	0	1	0	0	0
*O. kokonoricus*	19	0	1	0	0	0
*O. kokonoricus*	19	0	1	0	0	0
*O. kokonoricus*	16	0	1	0	0	0
*O. kokonoricus*	16	0	1	0	0	0
*O. kokonoricus*	16	0	1	0	0	0
*O. kokonoricus*	16	0	1	0	0	0
*O. kokonoricus*	16	0	1	0	0	0
*O. kokonoricus*	15	0	1	0	0	0
*O. kokonoricus*	15	0	1	0	0	0
*O. kokonoricus*	15	0	1	0	0	0
*O. kokonoricus*	15	0	1	0	0	0
*O. kokonoricus*	7	0	1	0	0	0
*O. kokonoricus*	7	0	1	0	0	0
*O. kokonoricus*	7	0	1	0	0	0
*O. kokonoricus*	7	0	1	0	0	0
*O. kokonoricus*	7	0	1	0	0	0
*O. intermedius*	22	0	0.99999	0	0	0.00001
*O. intermedius*	22	0	1	0	0	0
*O. intermedius*	22	0	1	0	0	0
*O. intermedius*	22	0	1	0	0	0
*O. intermedius*	23	0	1	0	0	0
*O. intermedius*	23	0	0.99918	0	0	0.00082
*O. intermedius*	23	0	1	0	0	0
*O. intermedius*	23	0	1	0	0	0
*O. intermedius*	23	0	0.99999	0	0	0.00001
*O. intermedius*	20	0	1	0	0	0
*O. intermedius*	20	0	1	0	0	0
*O. intermedius*	20	0	1	0	0	0
*O. intermedius*	20	0	1	0	0	0
*O. intermedius*	25	0	1	0	0	0
*O. intermedius*	25	0	1	0	0	0
*O. intermedius*	25	0	1	0	0	0
*O. intermedius*	25	0	1	0	0	0
*O. intermedius*	25	0	1	0	0	0
*O. intermedius*	26	0	1	0	0	0
*O. intermedius*	26	0	1	0	0	0
*O. intermedius*	26	0	1	0	0	0
*O. intermedius*	26	0	1	0	0	0
*O. intermedius*	26	0	1	0	0	0
*O. intermedius*	28	0	0.88592	0	0	0.11408
*O. intermedius*	28	0	0	0	0	1
*O. intermedius*	28	0	0.00012	0	0	0.99988
*O. intermedius*	28	0	0	0	0	1
*O. intermedius*	21	0	1	0	0	0
*O. intermedius*	21	0	1	0	0	0
*O. intermedius*	21	0	1	0	0	0
*O. intermedius*	24	0	0.99743	0	0	0.00257
*O. intermedius*	24	0	1	0	0	0
*O. intermedius*	24	0	1	0	0	0
*O. intermedius*	24	0	1	0	0	0
*O. intermedius*	27	0	0	0	0	1
*O. intermedius*	27	0	0	0	0	1
*O. intermedius*	27	0	0	0	0	1
*O. intermedius*	27	0	0	0	0	1
*O. intermedius*	27	0	0	0	0	1
*O. thoroldii*	29	0.11855	0	0	0.88145	0
*O. thoroldii*	29	1	0	0	0	0
*O. thoroldii*	29	0.00002	0	0	0.99998	0
*O. thoroldii*	29	0.95236	0	0	0.04764	0
*O. thoroldii*	30	0.98688	0	0	0.01312	0
*O. thoroldii*	30	1	0	0	0	0
*O. thoroldii*	30	0.00092	0	0	0.99908	0
*O. thoroldii*	30	0.99997	0	0	0.00003	0
*O. thoroldii*	30	0.94228	0	0	0.05772	0
*O. thoroldii*	31	1	0	0	0	0
*O. thoroldii*	31	1	0	0	0	0
*O. thoroldii*	31	1	0	0	0	0
*O. thoroldii*	31	1	0	0	0	0
*O. thoroldii*	31	1	0	0	0	0
*O. thoroldii*	33	1	0	0	0	0
*O. thoroldii*	33	1	0	0	0	0
*O. thoroldii*	33	0.99999	0	0	0.00001	0
*O. thoroldii*	33	1	0	0	0	0
*O. thoroldii*	33	1	0	0	0	0
*O. thoroldii*	37	1	0	0	0	0
*O. thoroldii*	37	1	0	0	0	0
*O. thoroldii*	37	1	0	0	0	0
*O. thoroldii*	37	1	0	0	0	0
*O. thoroldii*	37	1	0	0	0	0
*O. thoroldii*	39	1	0	0	0	0
*O. thoroldii*	39	1	0	0	0	0
*O. thoroldii*	39	1	0	0	0	0
*O. thoroldii*	39	1	0	0	0	0
*O. thoroldii*	41	1	0	0	0	0
*O. thoroldii*	41	1	0	0	0	0
*O. thoroldii*	41	1	0	0	0	0
*O. thoroldii*	41	1	0	0	0	0
*O. thoroldii*	41	1	0	0	0	0
*O. thoroldii*	42	1	0	0	0	0
*O. thoroldii*	42	1	0	0	0	0
*O. thoroldii*	42	1	0	0	0	0
*O. thoroldii*	42	1	0	0	0	0
*O. thoroldii*	42	1	0	0	0	0
*O. thoroldii*	43	1	0	0	0	0
*O. thoroldii*	43	1	0	0	0	0
*O. thoroldii*	43	1	0	0	0	0
*O. thoroldii*	43	1	0	0	0	0
*O. thoroldii*	43	1	0	0	0	0
*O. thoroldii*	44	1	0	0	0	0
*O. thoroldii*	44	1	0	0	0	0
*O. thoroldii*	44	1	0	0	0	0
*O. thoroldii*	44	1	0	0	0	0
*O. thoroldii*	44	1	0	0	0	0
*O. thoroldii*	45	1	0	0	0	0
*O. thoroldii*	45	1	0	0	0	0
*O. thoroldii*	45	1	0	0	0	0
*O. thoroldii*	45	1	0	0	0	0
*O. thoroldii*	45	1	0	0	0	0
*O. thoroldii*	46	1	0	0	0	0
*O. thoroldii*	46	1	0	0	0	0
*O. thoroldii*	46	1	0	0	0	0
*O. thoroldii*	46	1	0	0	0	0
*O. thoroldii*	46	1	0	0	0	0
*O. thoroldii*	47	1	0	0	0	0
*O. thoroldii*	47	1	0	0	0	0
*O. thoroldii*	47	1	0	0	0	0
*O. thoroldii*	47	1	0	0	0	0
*O. thoroldii*	47	1	0	0	0	0
*O. thoroldii*	48	1	0	0	0	0
*O. thoroldii*	48	1	0	0	0	0
*O. thoroldii*	48	1	0	0	0	0
*O. thoroldii*	48	1	0	0	0	0
*O. thoroldii*	48	1	0	0	0	0
*O. thoroldii*	40	1	0	0	0	0
*O. thoroldii*	40	1	0	0	0	0
*O. thoroldii*	40	1	0	0	0	0
*O. thoroldii*	40	1	0	0	0	0
*O. thoroldii*	40	1	0	0	0	0
*O. thoroldii*	38	1	0	0	0	0
*O. thoroldii*	38	1	0	0	0	0
*O. thoroldii*	38	1	0	0	0	0
*O. thoroldii*	38	1	0	0	0	0
*O. thoroldii*	38	1	0	0	0	0
*O. thoroldii*	36	1	0	0	0	0
*O. thoroldii*	36	1	0	0	0	0
*O. thoroldii*	36	1	0	0	0	0
*O. thoroldii*	36	1	0	0	0	0
*O. thoroldii*	36	1	0	0	0	0
*O. thoroldii*	34	1	0	0	0	0
*O. thoroldii*	34	1	0	0	0	0
*O. thoroldii*	34	1	0	0	0	0
*O. thoroldii*	34	1	0	0	0	0
*O. thoroldii*	34	1	0	0	0	0
*O. thoroldii*	35	1	0	0	0	0
*O. thoroldii*	35	1	0	0	0	0
*O. thoroldii*	35	1	0	0	0	0
*O. thoroldii*	35	1	0	0	0	0
*O. thoroldii*	35	1	0	0	0	0
*O. thoroldii*	32	1	0	0	0	0
*O. thoroldii*	32	1	0	0	0	0
*O. thoroldii*	32	1	0	0	0	0
*O. thoroldii*	32	1	0	0	0	0
*O. thoroldii*	32	1	0	0	0	0
